# An Integrated Virtual Reality–Based Telerehabilitation Platform to Support Recovery and Maintenance of Functional Abilities Among Older Adults: Protocol for a Usability and Acceptability Study

**DOI:** 10.2196/68358

**Published:** 2025-07-29

**Authors:** Marco Benadduci, Claudia Franceschetti, Rachele Alessandra Marziali, Sebastian Frese, Peter Stephan Sándor, Valentina Tombolesi, Valentina Bozzi, Lorena Rossi

**Affiliations:** 1 Centre for Innovative Models for Aging Care and Technology IRCCS INRCA Ancona Italy; 2 Technology and Innovation Unit and Research Department ZURZACH Care Bad Zurzach Switzerland; 3 Neurorehabilitation and Research Department ZURZACH Care Bad Zurzach Switzerland; 4 Scientific Direction IRCCS INRCA Ancona Italy

**Keywords:** rehabilitation, upper limb, stroke, traumatic brain injury, immersive virtual reality, virtual reality, home care, asynchronous telerehabilitation

## Abstract

**Background:**

Population aging leads to increased disability, implying a significant effect on health care systems and the lives of caregivers. As an example, stroke is a major cause of common diseases and is one of the leading causes of disability in older adults. Rehabilitation is the most effective intervention to counteract patient disability and simultaneously reduce the burden on caregivers. In particular, repetitive and task-specific training seems to be the most effective intervention for poststroke rehabilitation. Virtual reality (VR) is a very useful tool to provide this type of intervention, making it fun through gamification.

**Objective:**

This paper aims to present a protocol to evaluate the acceptability and usability of an upper limb rehabilitation solution based on VR. The RecoveryFun telerehabilitation system consists of a VR headset, wearable sensor, caregiver app, and clinical platform.

**Methods:**

A total of 15 older adult patients with neurological conditions (eg, stroke or brain injury) fulfilling the inclusion and exclusion criteria were recruited in 3 recruitment centers, 5 from each site. The system was given to patients, and they were free to use it when they preferred at their home, with or without caregiver help, following the clinical session set by the physiotherapist. At least 20 minutes of use per week was requested. The physical therapist was able to remotely monitor the progress of the therapy and increase the difficulty and repetitions of the exergames, also considering the patient’s fatigue and stress levels. The system was kept by the patients for 4 weeks, and there were several meetings and supervision via phone calls by the therapist. The main dimensions investigated were system usability, using the System Usability Scale (SUS) and User Experience Questionnaire, and acceptability, using the Quebec User Evaluation of Satisfaction with Assistive Technology (QUEST) 2.0. Upper limb function and patient’s quality of life, as well as caregiver’s perceived stress, were also assessed as secondary outcomes.

**Results:**

The study started in May 2024 and ended in June 2024. We recruited 16 patients with their caregivers and 6 health care professionals in Italy, Belgium, and Switzerland. Results are expected to be published by winter 2025.

**Conclusions:**

The aim of the study is to propose and evaluate a new telemedicine system that would allow greater adherence to therapy without moving from home, reducing the burden on the caregiver. The system could also be used in rehabilitation centers as a complement to traditional rehabilitation. Finally, with the calibration system enabling the therapist to create customized clinical sessions for the patient, the system could be versatile and fun for a wide range of patients.

**Trial Registration:**

ClinicalTrials.gov NCT06640452; https://clinicaltrials.gov/study/NCT06640452

**International Registered Report Identifier (IRRID):**

RR1-10.2196/68358

## Introduction

Aging is characterized by a progressive decline in physiological integrity, leading to impaired functional ability and ultimately increased mortality [1]. This decline is partially linked to an increased risk of chronic diseases, including cardiovascular diseases, diabetes, neurological disorders, and cancer. Furthermore, individuals with chronic diseases, like multiple sclerosis, are also exposed to the decline in physiological integrity associated with aging. Additionally, age-related musculoskeletal disorders like arthritis, osteoporosis, and frailty are associated with pain, mobility disorders, an increased risk of falls and fractures, and impaired ability or even disability to perform activities of daily living [2].

Age-related diseases not only cause poorer health and disability in individuals but also significantly impact the lives of caregivers, increasing their stress due to the heightened caregiving burden [3]. Moreover, age-related diseases represent a burden on health care systems and economies through loss of productivity and high health care costs [4-6]. Thus, the aging population is challenging health care systems to ensure long-term rehabilitation for patients [7].

Given the increasing burden of age-related and neurological diseases, developing accessible, cost-effective rehabilitation methods is critical for improving health care outcomes globally.

In particular, the global prevalence of neurological diseases has risen sharply with the significant aging of the global population in recent years [8]. Notably, stroke is the third leading cause of long-term disability in most countries and can result in paralysis, speech impairment, loss of memory and reasoning ability, coma, or even death [9]. Additionally, acquired brain injuries, such as traumatic brain injury, can lead to similar long-term disabilities, including balance and motor impairments, cognitive complaints related to memory and concentration, and chronic headache [10].

The most commonly reported impairment after a stroke or acquired brain injury is the complete or partial loss of motor function in the upper extremities, which can hinder activities of daily living and significantly undermine the quality of life of patients [9].

However, recent studies indicate that highly repetitive, task-specific, and sufficiently challenging training may promote neuroplastic changes to restore motor function [11]. Hence, virtual reality (VR) exergaming has gained attention in rehabilitation, as it incorporates key elements of neuroplasticity (ie, feedback, repetition, intensity, and task specificity, leading to potential recovery in the upper extremities) [12].

VR-based interventions further support cortical reorganization by engaging patients in meaningful, goal-directed activities that stimulate neuroplasticity [13]. Combining physical and cognitive tasks, VR exergaming activates sensorimotor and cognitive networks, which may strengthen neural pathways and enhance functional recovery after stroke or brain injury [14].

Moreover, growing evidence suggests that VR exergaming can be effectively used in the context of telerehabilitation, as VR exergaming offers the possibility to generate individualized, safe, and multimodal simulations that have the potential to create a location-independent, effective rehabilitation environment [15]. This approach allows patients to transfer a part of their rehabilitation services from the clinical setting to their home environment [16]. Such a shift provides several advantages, including making rehabilitation programs more adaptable to patients’ schedules, partially alleviating the time constraints experienced by therapists, reaching geographically isolated areas without clinical facilities, and reducing costs (eg, by decreasing travel to rehabilitation clinics or enabling home visits) [17,18].

In light of this, research regarding the usability and effectiveness of VR in rehabilitation has grown recently. However, the main application of VR to date is aimed at increasing balance [19]. The use of VR in upper limb rehabilitation can be a viable telerehabilitation solution that encourages patients to perform therapy when they want at home. To date, telerehabilitation systems for upper limb rehabilitation are limited to the use of semi-immersive rather than fully immersive reality, like is provided by RecoveryFun [20].

To address this gap, we developed RecoveryFun—an innovative motor and cognitive telerehabilitation system based on immersive VR and Internet of Things (IoT) targeted at patients with neurological conditions (eg, stroke or brain injury) with upper limb impairments. The RecoveryFun solution assists health care professionals with creating personalized rehabilitation plans, allowing them to customize exercises by type and difficulty level. Patients can perform these exercises comfortably at home or in the clinic using the provided VR headset. Through a clinical portal, health care personnel can monitor adherence to the rehabilitation plan, track progress, and make timely adjustments as needed.

Despite the growing interest in such systems, the market still lacks comprehensive, integrated VR telerehabilitation platforms. For this reason, this study will assess the usability and acceptance of the RecoveryFun prototype through practical functional testing of the system, games, and caregiver app.

## Methods

### Overview

The study was conducted using a pre-post approach, with a group of patients receiving the system and undergoing data collection before and after the installation and use of the technical solution. Three centers of recruitment were involved: Italian National Institute of Health and Science on Aging (IRCCS INRCA; Italy), Trainm (Belgium), and ZURZACH Care (Switzerland). Each center had to recruit 5 patients with their caregivers. A total of 30 participants had to take part in the trial, excluding the physiotherapists. The study lasted 4 weeks, during which time each participant followed an individualized rehabilitation plan of at least two rehabilitation sessions ranging from a minimum of 10 minutes to a maximum of 30 minutes per week. Each rehabilitation session was planned and scheduled by the therapist remotely via the dedicated clinical platform. The physiotherapist, by monitoring performance and adherence data regarding previous sessions, was able to create sessions customized to the patient by choosing the most useful level and exergames according to the rehabilitation goals. The patient could still perform more sessions if desired.

### The RecoveryFun System

#### Overview

The RecoveryFun system is represented by a series of integrated components, which will be presented in detail in this section.

The patient is provided with a VR headset with the exergame VR application to perform his or her training session, a wearable sensor that connects to the network through a gateway to monitor physical parameters during training sessions, a tablet for remote support from the clinical staff, and a router to provide an internet connection.

The informal carer is able to monitor, support, and motivate the patient’s activity using the mobile carer app. He or she also has the possibility to use the tablet to visualize the VR environment in which the patient is playing.

The professionals (carer, therapist, and management staff) rely on the clinical platform to manage the clinical session settings and monitor the patient’s data, with a dedicated decision support system.

**Figure 1:**
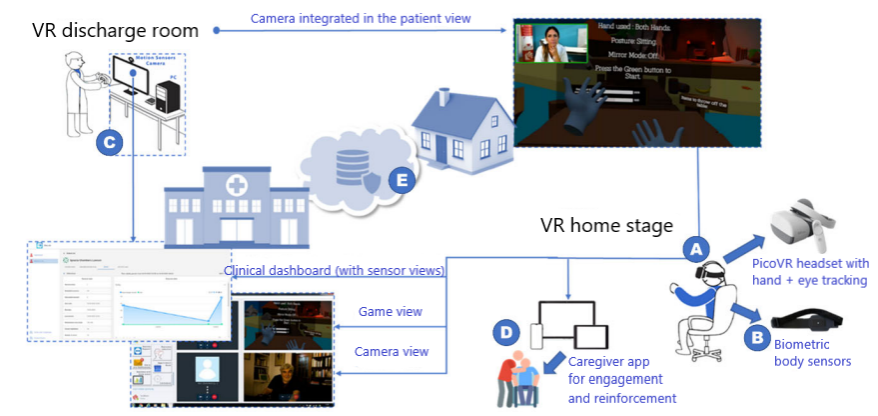
RecoveryFun system architecture: (A) virtual reality (VR) headset, (B) wearable sensor, (C) clinical platform, (D) caregiver app, (E) smart services.

The RecoveryFun system architecture is represented in Figure 1.

#### VR Headset

**Figure 2:**
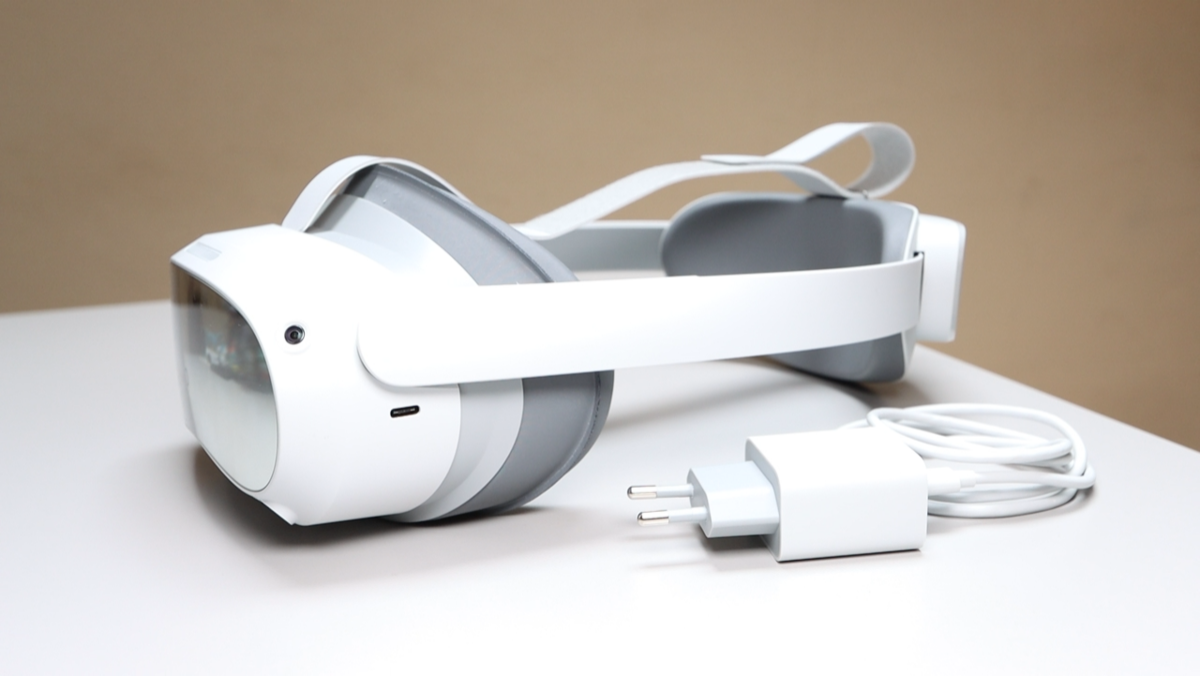
Virtual reality headset.

The RecoveryFun exergame app developed for rehabilitation purposes is implemented on the PICO 4 VR headset (Figure 2). The device represents the latest version available on the market from PICO at the time of system development. In addition to eye tracking, this VR headset is equipped with hand tracking functionality, which makes it easily usable by patients wearing biosensors during the training session.

#### Exergames

A set of exergames is implemented in the RecoveryFun app for rehabilitation purposes. In particular, they are designed to improve and maintain upper limb functionality and to stimulate cognitive exercises. They are developed with special attention to the spatial anchoring of patients, as a measure to increase acceptance and reduce the risk of dizziness. For each exergame, the motion parameters are based on the measurements evaluated in the calibration phase performed during the first session with the physiotherapist. The calibration consists of a series of activities performed by the patient in the VR environment that serve to assess hand motricity, the space that can be explored by the upper limb, and reaction time, in order to adapt the exergames to the patient’s specific needs and abilities. Activities performed by the patient to investigate these motor dimensions include opening and closing the hand, moving the arm in the frontal space, and hitting objects that appear in the explored space.

The system includes 7 different exergames, as described in the following paragraphs.

**Figure 3:**
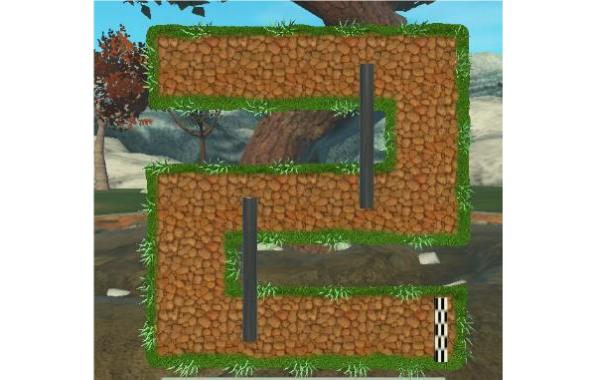
Escape from Alpatraz exergame.

In e*scape from Alpatraz* (Figure 3), the patient has to grab an alpaca and drag it to the final line of the maze. The game stimulates eye motor coordination skills and offers a cognitive exercise in which the patient performs forced movements to reach the target. Motor control and proprioception of the upper limb are trained. This exercise improves muscle endurance, by requiring the patient to keep the arm elevated throughout the length of the line. In addition, in the most difficult level of the game, the patient must be able to recognize the correct line.

**Figure 4:**
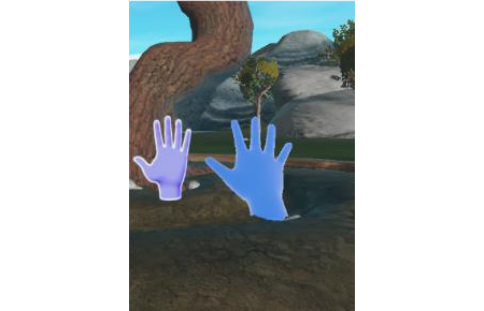
Clapping hands exergame.

In *clapping hands* (Figure 4), the person has to imitate the gesture he or she sees then push his or her hand toward the image. The game stimulates the ability to reproduce observed gestures. All joints of the upper limb are trained. This exercise can also be useful if the patient has ideomotor apraxia.

**Figure 5:**
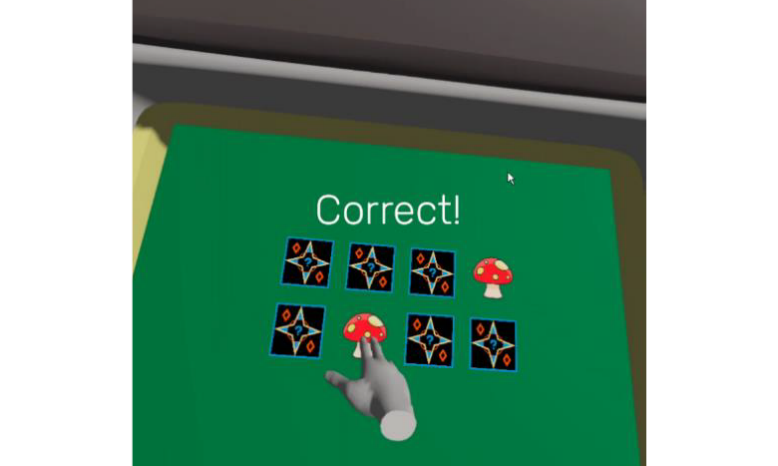
Memory exergame.

*Memory* (Figure 5) requires the person to find pairs of similar images. It stimulates the user’s memory and involves moving all the upper limbs. Since this exercise requires precise movements, patients with dysmetria may benefit from it.

**Figure 6:**
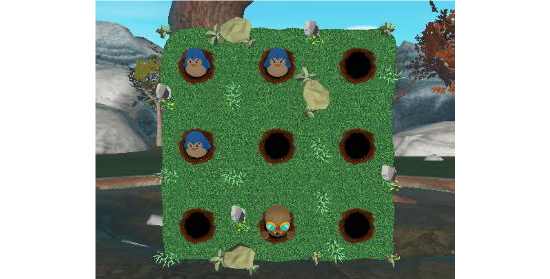
Whack a mole exergame.

In *whack a mole* (Figure 6), the person has to hit moles with their hand and avoid the penguins. The game stimulates the patient’s reaction time and requires flexing the shoulder and extending the elbow to reach the target. The patient has to quickly differentiate between the penguins and moles.

**Figure 7:**
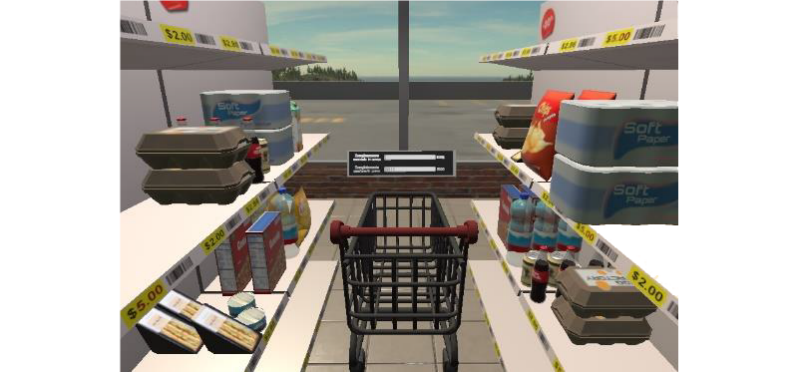
Groceries exergame.

*Groceries* (Figure 7) involves the person putting only the listed products into the shopping cart. At the start of the game, a shopping list is shown, and the person has to memorize it. The game trains skills such as spatial exploration (the patient has to move their head to find all the items) and memory. This exercise is repetitive and task-oriented, providing an effective approach for stroke recovery [10].

**Figure 8:**
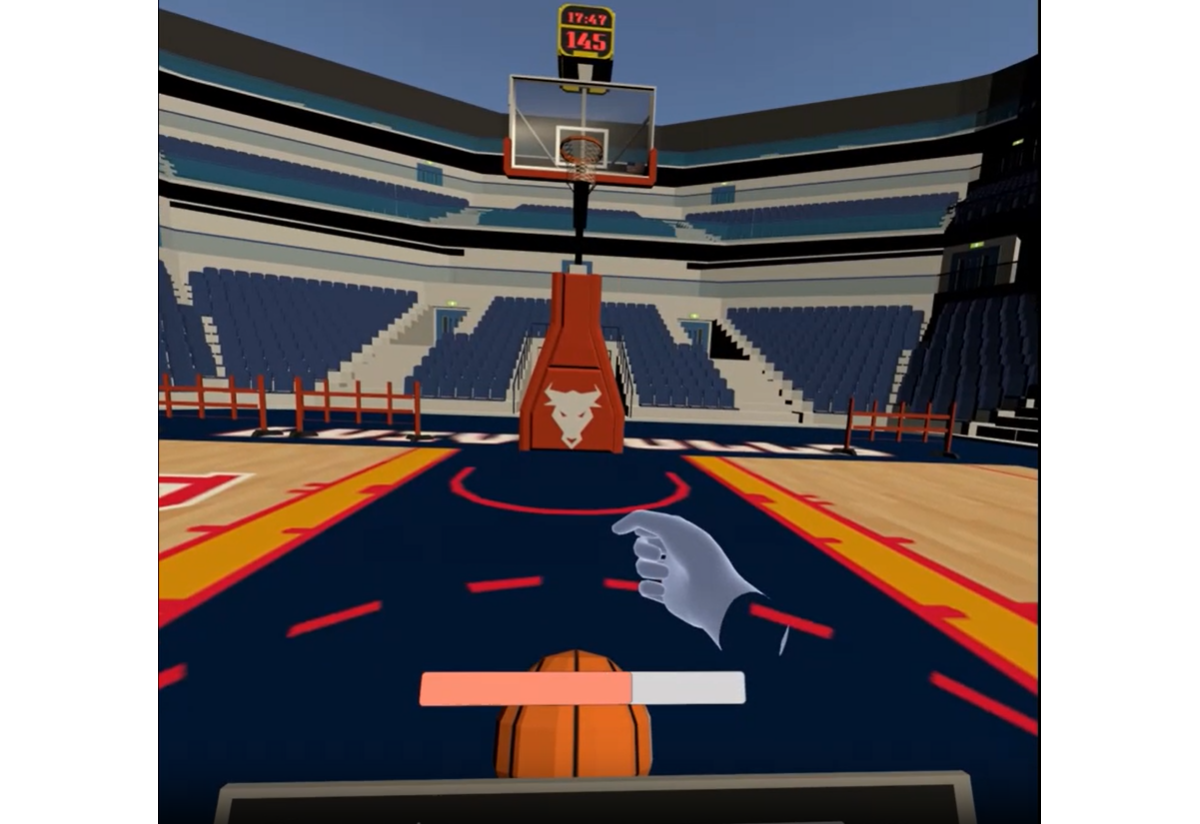
Basket exergame.

In *basket* (Figure 8), the person has to close his or her hand in a fist to charge the power of the shot (loading bar). When the patient fully loads the bar, he or she has to open the hand to score. The game trains finger flexion and coordination

**Figure 9:**
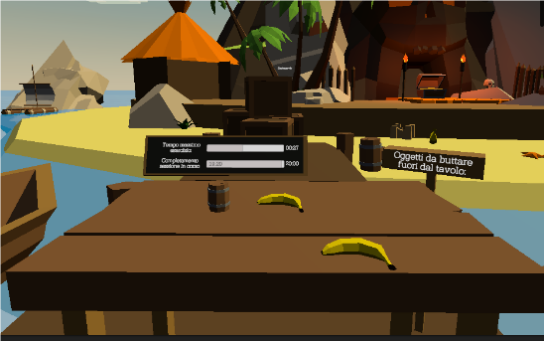
Table trouble exergame.

*Table trouble* (Figure 9) requires the person to throw specific objects into the water. The patient has to move their arm across the entire field of view and in all spatial planes. This exercise can be particularly useful for patients with associative agnosia.

#### Wearable Sensor

**Figure 10:**
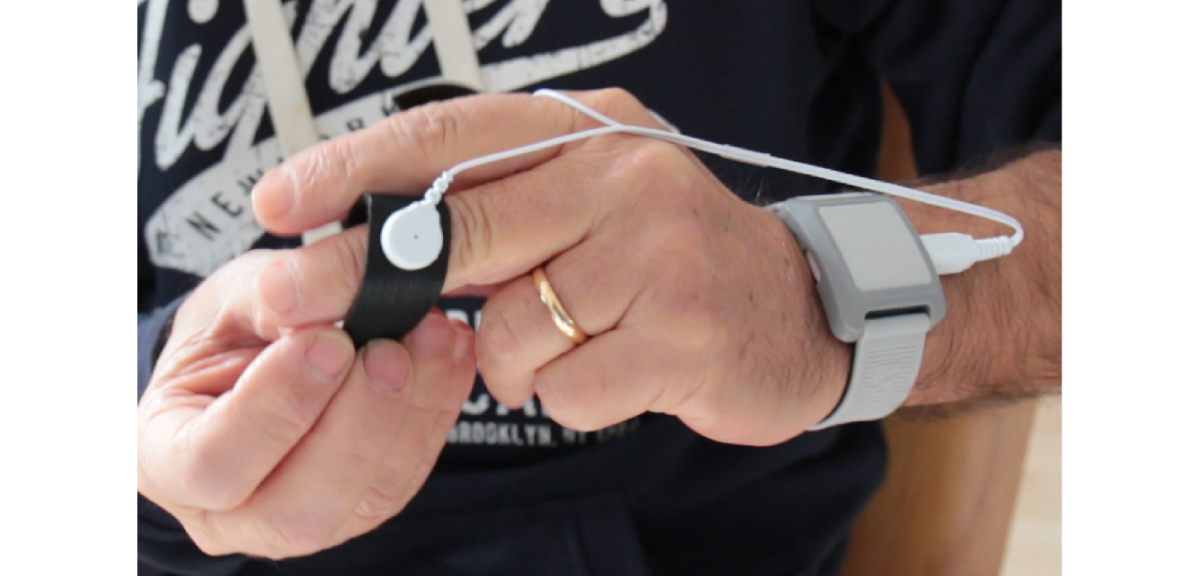
Wearable sensor.

A biosensing wireless sensor (wristband), shown in Figure 10, is integrated in the IoT connected ecosystem to measure physiological parameters (photoplethysmography and galvanic skin response data) of end users during the rehabilitation session. The collected data are then processed by an appropriate algorithm to derive measures of stress and fatigue for clinicians to review.

#### Caregiver App

**Figure 11:**
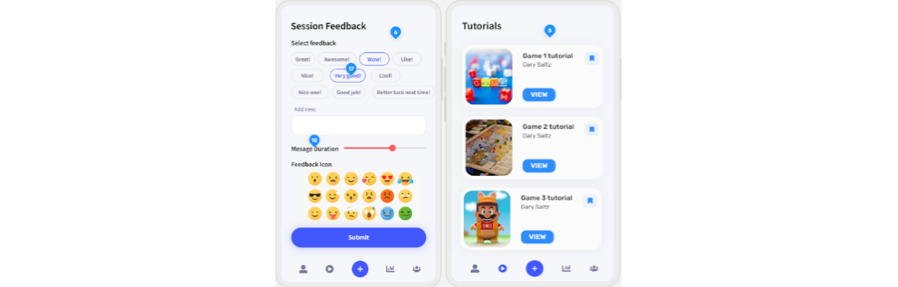
Example caregiver app screens.

The informal carer has access (only) to the secure mobile caregiver app installed on his or her smartphone (Figure 11). Its main goal is to enable the caregiver to motivate and support the end user with therapy adherence. No medical data are presented in the caregiver app. Moreover, motivational messages sent by the caregiver from this app are visible to the patient on the RecoveryFun VR app’s home screen.

Using the app, the caregiver can monitor the patient’s training adherence; send motivational messages to the patient, who will see them on the RecoveryFun VR app’s home screen; and consult support material on the use of the system (explanatory videos and a frequently asked questions [FAQ] section) to help the patient use the devices, acting as first-level assistance.

#### Clinical Platform

**Figure 12:**
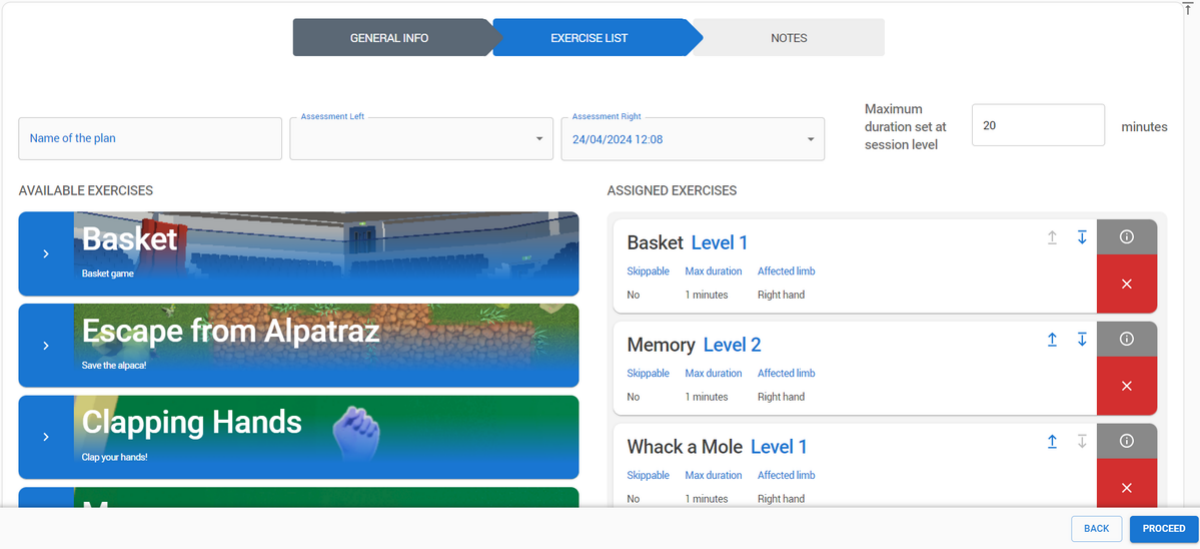
Clinical platform screen.

Clinical personnel have access to the clinical platform provided as a secure web application (Figure 12). The system administrator can control access rights for each clinical user group (eg, doctors, physiotherapists, nurses).

In the clinical platform, an authorized clinician can edit the clinical and administrative data for the assigned patients, manage the VR devices and assign them to a specific patient, associate the caregiver with the corresponding patient, create rehabilitation plans for specific patients, monitor the activity of patients (ie, observe the progress of the rehabilitation sessions executed, see the number and the duration of training sessions executed, evaluate compliance to the rehabilitation plan), and connect to a secure video channel to see the patients’ activities in the VR environment.

#### Smart Services

The platform in the future will also include the presence of smart services, which are under development. This component of the system aims at improving clinicians’ activities, allowing for further customization of the rehabilitation experience for the patient and providing a decision support system for the clinical staff.

### The Field Trial

The field trial procedure was divided into different phases. Table 1 describes each of them.

**Table 1:** Field trial phases.

Phase number	Phase name	Description
1	Recruitment, baseline evaluation, education sessions, and calibration	This phase first involved the recruitment assessment to ensure the patient met the inclusion and exclusion criteria and the baseline assessment with the patient and caregiver to collect their data. The first education session showed the patient and caregiver how to operate and properly use the system. The number of possible further education sessions was then planned based on the patient’s and caregiver’s confidence using the system independently at home. Finally, the calibration phase was executed if the patient agreed to participate in the study.
2	System installation and use	After the conclusion of the education sessions, the system was installed at home, and the patient began rehabilitation sessions, with the support of the informal caregiver. The physiotherapist constantly monitored the treatment progress through the clinical platform available to him or her.
3	Monitoring (at the end of each week)	The clinician called the patient weekly to ask how he or she was doing, how the sessions were going, and whether there were any difficulties or technical problems. If the problem could not be solved remotely, a meeting was scheduled at home or in the hospital.
4	Final evaluation (after 4 weeks of use)	The aim of this phase was to assess the usability and acceptability of the system and document any critical issues detected by the patient, to evaluate the perceived stress and usability of the system by the informal caregiver, and, finally, to collect information on the impact and usability of the system according to health professionals.

### Recruitment

The population selected for the trial in the RecoveryFun project consisted of patients who had overcome an acute event and completed intensive inpatient rehabilitation. The patient we were referred to had already returned home and needed to maintain goals already achieved or increase functionality.

For the patient to be able to use the system properly (to date), specific physical and cognitive requirements are needed: The patient has to have good trunk control and be able to maintain a sitting position for at least 30 minutes without feeling fatigue, and he or she must have good upper limb function (gross and fine hand motor skills). Even when the game workspace is defined according to patient characteristics during the calibration phase, the shoulder range of movement must be wide enough to be able to reach the boundary of the minimum definable workspace. Moreover, the patient has to be able to transfer load onto the pelvis.

In addition, the patient should have a caregiver who, if necessary, can assist him or her during the game session (wearing the headset and the wearable sensor, initiating the games, and stimulating him or her).

The following activities were carried out during recruitment: patient and caregiver information and informed consent, verification of inclusion and exclusion criteria, collection of medical history, objective examination, administration of rating scales, and collection of patient and caregiver sociodemographic data.

### Patients

Once the informed consent was obtained in duplicate, compliance with the study’s inclusion and exclusion criteria was verified, and the baseline evaluation was carried out with the questionnaires and clinical trials provided by the study design.

Patient inclusion criteria were age ≥60 years; no more than 12 months from an acute event (eg, stroke or brain injury); Trunk Impairment Scale score ≥20; presence of an available caregiver during the study, and the following for the Fugl-Meyer Assessment (FMA) Upper Extremities scale: score ≥33 for motor function of the upper extremities (sections A-D) as well as (Section A.II. Volitional movement) values of 1 or 2 for shoulder elevation, shoulder abduction (90°), external shoulder rotation, elbow flexion and extension, and forearm pronation; (Section B. Wrist) values of 1 or 2 for items rating stability at 15° dorsiflexion (elbow at 90° and at 0°); (Section C. Hand) values of 1 or 2 for pincer grasp, pincer opposition, and cylinder grasp; and (Section D. Coordination/speed) a value of 1 or 2 for tremor. Sections H (Sensation) and J (Passive joint motion and Joint pain) were not considered, as these parameters do not influence the participants’ capacity to participate in the intervention.

Patient exclusion criteria were an inability to give his or her informed written consent; presence of cognitive impairment (Montreal Cognitive Assessment score <24 [21]); not meeting the technical requirements based on the safety manual of the PICO headset (eg, having ocular pathologies such as cataracts, glaucoma, and diabetic retinopathy; binocular vision abnormalities; a high degree of myopia, astigmatism, or far-sightedness; corrective glasses that do not fit underneath the VR headset; allergy to plastic, polyurethane, or fabric; a pacemaker; an implanted defibrillator; or cochlear implants and other hearing aids; inability to independently put the VR goggles on); and the presence of a pathology that could impact the ability to use the VR system or be worsened by using the VR system (ie, epilepsy; migraine especially with aura and tension headache; vertigo or cybersickness; trigeminal neuralgia; pressure sensitivity and hyperalgesia in the face; psychotic disorders; severe cardiac or pulmonary conditions; open wounds, injuries to the head, skin infections, and dermatological issues that would prohibit wearing the headset; tremor or severe fatigue and exhaustion so that the participant is unable to concentrate or stay awake and attentive long enough to participate in the intervention for 20 minutes; various phobias [eg, claustrophobia]; and visual neglect).

### Outcomes

The field trials aimed to assess the usability and acceptance of the RecoveryFun system and adherence to therapy. The primary outcomes of the study included usability perceived by the patient, assessed using the SUS [22] and UEQ [23]; usability perceived by the informal caregiver, assessed using the SUS [22]; and acceptance perceived by the patient, assessed using the Quebec User Evaluation of Satisfaction with Assistive Technology (QUEST) 2.0 [24].

The field trials also focused on the impact of the RecoveryFun system, as measured in the secondary outcomes. These included the impact of the system on the patient’s quality of life, measured with the World Health Organization Quality of Life (WHOQOL-BREF) [21]; patient’s functional ability, measured with the FMA [25]; stress perceived by the caregiver, assessed using the Perceived Stress Scale [26] and an ad hoc questionnaire; patient interest in using and attitude toward playing, assessed using an ad hoc questionnaire; and the impact of the system on the caregiver’s ability to understand whether it is a helpful and supportive tool or causes additional difficulty, as assessed using an ad hoc questionnaire.

For these reasons, the protocol includes study-specific questions on demographics and attitudes toward technology from both the patient and the informal caregiver. In addition, questions related to subjective health and social support are also explored with the patient.

Tables 2 and 3 summarize the different tools adopted with each end user group in all the phases of the study**.**

**Table 2:** Tools and dimensions of the patient protocol.

Dimension	Tool	Recruitment	Baseline (T0)	Final evaluation (T1)
Functional ability	FMA^a^ [25]	✓	—^b^	✓
Trunk stability	TIS^c^ [27]	✓	—	—
Cognitive status	MoCA^d^ [28]	✓	—	—
Sociodemographic characteristics; subjective health assessment; social support	Ad hoc questionnaire	—	✓	—
Attitude to technology	MPT-SOTU-C^e^ [29]	—	✓	—
Quality of life	WHOQOL-BREF^f^ [21]	—	✓	✓
Acceptance	QUEST^g^ 2.0 [24]	—	—	✓
Usability	SUS^h^ [22]	—	—	✓
Usability	UEQ^i^ [23]	—	—	✓
Intention to pay	Ad hoc questionnaire	—	—	✓

^a^FMA: Fugl-Meyer Assessment.

^b^Not applicable.

^c^TIS: Trunk Impairment Scale.

^d^MoCA: Montreal Cognitive Assessment.

^e^MPT-SOTU-C: Matching Person and Technology Survey of Technology Use-Consumer.

^f^WHOQOL-BREF: World Health Organization Quality of Life Brief

^g^QUEST: Quebec User Evaluation of Satisfaction with Assistive Technology.

^h^SUS: System Usability Scale.

^i^UEQ: User Experience Questionnaire.

**Table 3:** Tools and dimensions of the informal caregiver protocol.

Dimension	Tool	Recruitment	Baseline (T0)	Final evaluation (T1)
Sociodemographic characteristic	Ad hoc questionnaire	—^a^	✓	—
Attitude to technology	MPT-SOTU-C^b^ [29]	—	✓	—
Perceived stress	PSS^c^ [26]	—	✓	✓
Perceived stress	Ad hoc questionnaire	—	—	✓
Usability	SUS^d^ [22]	—	—	✓
Impact of the system	Ad hoc questionnaire	—	—	✓

^a^Not applicable.

^b^MPT-SOTU-C: Matching Person and Technology Survey of Technology Use-Consumer.

^c^PSS: Perceived Stress Scale.

^d^SUS: System Usability Scale.

In addition, during the course of the month, weekly phone calls or messages were conducted, in which patients could report any critical issues, such as difficulties with playing the game, technical problems, or any feelings of discomfort related to possible motion sickness. The researcher, if necessary, visited the patient’s home to solve the critical issues and make suggestions to improve the user experience.

### Statistical Analysis

The first step of the data analysis involved the description of the sample. Continuous variables were reported as either mean and SD or median and IQR based on their distribution (assessed using the Kolmogorov-Smirnov test). Categorical variables were expressed as absolute numbers and percentages. The comparisons between pre- and postintervention conditions were conducted using paired *t* tests (for normal distributions), Mann-Whitney *U* tests (for non-normal distributions), or chi-square tests (for categorical variables). Moreover, a linear regression model on the outcome variation between baseline and follow-up was estimated in order to evaluate measure variation.

A limitation of this analysis is the absence of formal adjustments for multiplicity due to multiple outcomes and repeated measures, which may increase the type I error rate. This choice was driven by the limited sample size, as this is a pilot study primarily aimed at assessing feasibility and generating preliminary data. Although basic statistical tests were appropriately applied, no correction methods (eg, Bonferroni adjustment, false discovery rate control, or hierarchical testing procedures) were implemented, as such adjustments would substantially reduce statistical power in this context. Future larger-scale studies should incorporate appropriate multiplicity control strategies to ensure more rigorous error rate control and improve the robustness of the statistical inferences.

### Dropouts and Replacement

When the physiotherapist noticed anomalies in the clinical platform that could be related to patient difficulties (eg, lack of use, numerous errors, or abandoned sessions), it was his or her responsibility, during the weekly monitoring, to investigate these aspects in order to keep the patient in the study.

If the patient expressed a wish to abandon the program, he or she was, of course, free to do so, provided that the physiotherapist had ensured that the decision was not based on any solvable problem. Taking into account that the usability and acceptability of the system were evaluated and that an objective was to assess the impact of the system on patient quality of life, a 20% dropout rate was considered acceptable. Therefore, our strategy consisted of replacing the patient if the dropout occurred in the middle of the study (at 2 weeks). 

On the contrary, if the patient abandoned the study more than 2 weeks after the start, the researchers were required to note the reasons for leaving and to collect the data gathered up to that point (by the system, researchers, and physiotherapist), unless the patient specifically asked to cancel the data collection.

Providing for the replacement of participants who dropped out was desirable but not mandatory in this case.

To ensure the total number of patients in the project, at least 6 participants had to be recruited at each site.

### Ethical Considerations

The study was approved in Italy by the Territorial Ethics Committee of Marche Region (Prot. CET-M 2023 353). Based on Swiss (Federal Act on Research involving Human Beings [Human Research Act]) [30] and Belgian [31] law, there was no ethics approval required.

The respect of ethical principles was one of the guidelines of the project and in this study in particular, because it involved a particularly vulnerable group.

All procedures involving human participants were in accordance with the ethical standards of the Helsinki declaration 1964 and its later amendments or comparable ethical standards.

All potentially eligible participants received comprehensive information about the study. All participants and caregivers were asked to provide signed informed consent prior to participating and were informed that data will be retained for up to 10 years after the end of the study. Each signature was personally dated by each signatory, and the informed consent and any additional patient information were retained by the investigator. A signed copy of the informed consent and information sheet were given to each patient.

Participants had to consent to the processing of their personal data in anonymous and aggregate form, in accordance with EU Regulation 2016/679 [32] on the protection of individuals with regard to the processing of personal data and Legislative Decree No. 101/2018 - Provisions for the adaptation of national legislation to the provisions of the European Regulation 2016/679 [33].

Participants were informed their data may be examined by authorized personnel or by members of the relevant ethics committee and officials of the competent regulatory authorities.

Participants could leave the project at any time if they considered participation too burdensome or felt uncomfortable for any reason.

No compensation was provided for participating in the study.

## Results

The study was conducted from May 2024 through June 2024 in Italy, Switzerland, and Belgium. A total of 16 patients, 16 caregivers, and 2 health care professionals took part in the field trial. The data collected have been analyzed and are expected to be published by winter 2025. The study findings will be used for publication in peer-reviewed scientific journals and presentations in scientific meetings.

## Discussion

In the face of an aging population, we will inevitably see an increase in acute cerebrovascular events, which are among the leading causes of disability in Europe, in the coming years [26]. Moreover, we will see an increase in the population’s demand for health care services [27]. As a result, we can expect to observe an increased care burden on the part of families. Taking these facts into account, interventions are needed to relieve families of these burdens and try to ensure the highest possible level of care even in the long term.

In this regard, the main treatment following cerebrovascular events is physiotherapy, which allows the pursuit of hospitals’ primary goal: regaining balance and standing in order to reduce the workload and ensure basic activities of daily living for the patient [34]. In particular, rehabilitation of the upper limb needs more time, as the required movements are finer and more complex [35].

Within this framework, the RecoveryFun project aims to offer an answer to these growing needs. RecoveryFun makes use of VR, as it is now recognized to be effective and helpful during upper limb rehabilitation for stroke patients [36]. In fact, the system is designed to provide personalized and fun upper limb telerehabilitation for patients. The professional, through his or her dedicated portal, can constantly remotely monitor the patient and motivate him or her to perform the exercises, resulting in more efficient work. In addition, the caregiver can significantly contribute to the patient's activity by using the caregiver app. He or she can monitor the patient's adherence to the therapy schedule, which is visible on the calendar screen, and easily send motivational messages. The caregiver can also provide first-level assistance if the patient has difficulty using the system autonomously, with the support of informative content such as video tutorials and the FAQ section uploaded to the caregiver app, and even contact technical support through the dedicated section.

All phases of the project were focused on the needs of the user and his or her caregiver in order for the system to be usable at home without the presence of a professional. Prior to the start of the experimentation, several tests were performed during the co-creation phase that helped optimize the system to be as responsive as possible to the needs of the end user. These tests were performed with both potential patients and professionals in the field.

In fact, although some of the devices used were already on the market, such as the Pico 4 Enterprise visor, the sensor was a prototype model that was not yet marketable. The games, platform, and dedicated caregiver app were developed for the project and optimized for the project’s goal. The calibration of the viewer, which then conditions the professional’s choice of games, required special work by the technical partners as it was not initially planned but was requested by the clinical partners because they wanted to avoid potential patient frustration during use as much as possible. Pilot tests were also carried out prior to the start of the trial to test the system with patients and collect any critical issues that the user might encounter.

The design of the study, described in this paper, allowed the solution to be tested for a period of only 1 month, which was a sufficient period to evaluate the usability and acceptability of the system. In fact, the study included a final evaluation in which both quantitative and qualitative instruments were administered. In addition, during the month, weekly feedback was collected via phone calls or messages. The main challenges of this study were to investigate whether the patient, even with low familiarity with technology, used the system consistently throughout the month-long trial and whether motivation was created.

To make sure to provide the system to patients who are able to use it, the choice of inclusion criteria took much time and thought within the consortium. In fact, the use of immersive VR for telerehabilitation is completely innovative and, according to the state of the art, has never been used before. The main concern of the researchers was not to generate frustration in patients, so specific criteria were identified to select patients with good upper limb function, excellent trunk control, and physiological or mild cognitive impairment.

Although the study focused on usability, the consortium decided to redo the FMA and WHOQOL-BREF also at the end of the test to collect more data and determine whether there was a change in upper limb function and quality of life for patients. The choice was also made due to existing evidence suggesting improvements in upper limb function in gross motor skills examined with the FMA [37]. Similarly, caregivers were given the Perceived Stress Scale again to assess any changes in perceived stress. This would be useful data for a potential future study on the effectiveness of the system.

In fact, the system has great potential for future development and could be of great help to rehabilitation centers, even during hospitalization, to supplement traditional rehabilitation. This use in a “protected” environment could make patients feel more confident when using the system, thus motivating them to use the system to receive motor and cognitive stimulation even once they return home. Moreover, telerehabilitation technologies could have great cost benefits, even if cost is complex to calculate and varies from country to country. A meta-analysis and systematic review [38] has highlighted how telerehabilitation is an effective way to not only reduce costs but also provide an extra service to patients living in rural or isolated areas. The RecoveryFun system is intended to allow neuromotor stimulation to continue at home and enable a personalized program.

A limitation of the system is that patients who can use it must have very specific requirements that significantly reduce the eligible population. However, the system, being extremely versatile, could also be developed for other areas and for the treatment of other diseases while maintaining the telerehabilitation mode.

## Abbreviations

FAQ: frequently asked questions
FMA: Fugl-Meyer Assessment
IoT: Internet of Things
IRCCS INRCA: Italian National Institute of Health and Science on Aging
SUS: System Usability Scale
UEQ: User Experience Questionnaire
VR: virtual reality
WHOQOL-BREF: World Health Organization Quality Of Life Brief
