# Correction: An Integrated Virtual Reality–Based Telerehabilitation Platform to Support Recovery and Maintenance of Functional Abilities Among Older Adults: Protocol for a Usability and Acceptability Study

**DOI:** 10.2196/88023

**Published:** 2025-12-24

**Authors:** Marco Benadduci, Claudia Franceschetti, Rachele Alessandra Marziali, Sebastian Frese, Peter Stephan Sándor, Valentina Tombolesi, Valentina Bozzi, Lorena Rossi

**Affiliations:** 1 Centre for Innovative Models for Aging Care and Technology, IRCCS INRCA Ancona Italy; 2 Technology and Innovation Unit and Research Department, ZURZACH Care Bad Zurzach Switzerland; 3 Neurorehabilitation and Research Department, ZURZACH Care Bad Zurzach Switzerland; 4 Scientific Direction, IRCCS INRCA Ancona Italy

In “An Integrated Virtual Reality–Based Telerehabilitation Platform to Support Recovery and Maintenance of Functional Abilities Among Older Adults: Protocol for a Usability and Acceptability Study” [1] the authors noted two errors.

In the originally published manuscript, affiliation 1 appeared as follows:

Centre for Innovative Models for Aging Care and Technology, Istituto Nazionale di Riposo e Cura per Anziani, Ancona, Italy

In the corrected manuscript, affliation 1 appears as follows:

Centre for Innovative Models for Aging Care and Technology, IRCCS INRCA, Ancona, Italy

In the originally published manuscript, affiliation 4 appeared as follows:

Scientific Direction, Istituto Nazionale di Riposo e Cura per Anziani, Ancona, Italy

In the corrected manuscript, affliation 4 appears as follows:

Scientific Direction, IRCCS INRCA, Ancona, Italy

The corrections will appear in the online version of the paper on the JMIR Publications website, together with the publication of this correction notice. Because these were made after submission to PubMed, PubMed Central, and other full-text repositories, the corrected article has also been resubmitted to those repositories.
